# Assessing Lower Urinary Tract Symptoms in Women Practising Competitive Judo: Findings from a Cross-sectional Study

**DOI:** 10.1007/s00192-026-06523-9

**Published:** 2026-02-27

**Authors:** Aleksandra Saulicz, Mariola Saulicz, Edward Saulicz

**Affiliations:** 1 School of Public Health & Social Work, Queensland University of Technology (QUT), Kelvin Grove, Queensland Australia; 2 Institute of Physiotherapy and Health Sciences, The Jerzy Kukuczka Academy of Physical Education, Katowice, Poland

**Keywords:** Judo, Lower urinary tract symptoms, Pelvic floor

## Abstract

**Introduction and Hypothesis:**

High impact physical activity (PA) in sportswomen is a risk factor for the development of stress-induced urinary incontinence and potentially for other dysfunctions of the pelvic floor. This study was aimed at assessing the occurrence of lower urinary tract symptoms (LUTS) in professional female judo practitioners.

**Methods:**

This observational, cross-sectional study included a total of 88 women practising professional judo and 88 women practising other sports disciplines. Participants completed Core Lower Urinary Tract Symptom Score (CLSS) questionnaire to assess the condition of the lower urinary tract, along with sport-related data questionnaires and self-reported anthropometric measures.

**Results:**

Among 19.31% of women practising judo no LUTS were recorded and the majority of the remaining judo athletes (67.6%) indicated the occurrence of one or two symptoms. Urgency was the most common symptom (40.9%). Six of the 10 analysed LUTS were statistically significantly less frequent in female judo athletes and the average number of LUTS was statistically significantly lower in this group (95% CI 1.67–2.48 vs 2.9–3.94; *p* < 0.001). Severity of three symptoms (nocturia, incomplete emptying of the bladder and urethral pain) was statistically significantly lower in women practising judo and their overall CLSS score was statistically significantly lower (95% CI 1.96–3.1 vs 3.9–5.68; *p* < 0.001). These athletes were also more satisfied with the level of acceptance of the CLSS condition (95% CI 0.67–1.18 vs 1.19–1.84; *p* < 0.01).

**Conclusions:**

Compared to other disciplines, competitive judo is not a risk factor for increased LUTS; therefore, the level of health satisfaction with the condition of the lower urinary tract in women practising judo is high.

**Supplementary Information:**

The online version contains supplementary material available at 10.1007/s00192-026-06523-9

## Introduction

Professional sports carry the risk of health damage, which is mainly related to the occurrence of various injuries. The participation of women in sports is also increasingly indicated as a risk factor for pelvic floor dysfunction [1, 2]. The high-impact physical activity (PA) typical of many sports activates muscles surrounding the abdominopelvic cavity and causes increased intra-abdominal pressure, which leads to the modification of the pelvic floor function [3]. Frequent and prolonged increased intra-abdominal pressure potential overloads the perineum. According to some researchers, such a condition contributes to fatigue and the weakened contractile strength of the pelvic floor muscles (PFMs) [4, 5]. In contrast, others indicate that high-impact PA is not so much related to the strength of the contraction of the PFMs, as it is to the endurance of the muscles that make up the pelvic floor (PF) [6]. Regardless of the dilemma of whether the problem lies in the strength of contraction or in the endurance of these muscles, over time this can lead to progressive damage to the musculo-fascial-ligamentous support structures that make up the PF [7]. The fact that the endopelvic fascia of the anterior vaginal wall, its connections to the arcus tendineus fascia pelvis and the medial portion of the levator ani muscles must remain intact to provide normal urethral support is of importance in this mechanism [8]. The damage to these structures directly disrupts the physiological mechanism of urination. The change in anatomical relationships and the natural level of passive tension lead to a change in the synergy between the abdominal muscles and the pubo-rectal muscles of the PF. This disturbs the functional balance between them, reciprocally resulting in a further loss of flexibility in the soft tissues that form the PF and consequently leads to a reduced tolerance to stretching [9]. Some authors point to the role of type II muscle fibres, which are characterised by a low capacity to maintain tension for a long time under such altered anatomical conditions, which promotes the further development of the functional failure of the PF [10–12]. An acquired dysfunction of these muscles initially results in sensory urgency and detrusor overactivity, which over time can lead to an acquired voiding dysfunction that can result in intermittent urine flow and incomplete bladder emptying [8]. Chronic damage of the perineum due to a frequent increase in intra-abdominal pressure during high-intensity PA with a weakened PF increases the risk of urinary loss during exercise (stress urinary incontinence—SUI) [2, 10, 13, 14].

Although sportswomen do not perceive urinary symptoms as a particularly pathological condition, these symptoms are of interest to numerous researchers because of the discomfort that may accompany them and their potential impact on athletic performance. They are associated with incidents of SUI during exercise, the prevalence of which among sports athletes is estimated to be 41–48% [15–19].

In the available literature, there are practically no studies on LUTS among women athletes practising combat sports. Judo is characterised by intense physical activity based on both dynamic and static muscle work. In the latter, sustained isometric muscle tension occurs while maintaining a grip or attempting to release it. This condition is associated with frequent and prolonged increases in intra-abdominal pressure, including the abdominal cavity. As previously mentioned, a rapid increase in intra-abdominal pressure is considered a cause of SUI in women undertaking high-intensity physical activity. It is well known that weight categories are an important element of judo. Reducing weight before the competition may have a negative impact on the immune system [20]. This is caused by the applied weight loss methods, which are a combination of food depravation, increased training volume and increased thermal stress [21–23]. Intense training sessions connected with an increase in intensity during this period lead to overtraining, which is manifested by an immune response [22]. Decreased immunity in turn heightens susceptibility to infection. Bladder pain and urethral pain are often associated with lower urinary tract infections. Judo’s specificity potentially poses some risk to the proper functioning of the lower urinary tract in women. This raises questions about the risk of urinary incontinence incidents and the frequency and quality of voiding. Another question concerns the occurrence of urethral and bladder pain.

The aim of this study is therefore to assess the incidence of lower urinary tract symptoms (LUTS) in women practising judo competitively compared with those practising other sports disciplines.

## Materials and Methods

This was an observational, cross-sectional study conducted in Poland, in sports clubs with a judo section and at the training camps of female athletes covered by a central training system related to appointments to the national team. The women in the control group were also recruited in sport clubs and training camps.

All study procedures were performed according to the Helsinki Declaration of Human Rights of 1975, modified in 1983. All participants gave their consent to participate after being informed of the study objectives and procedures.

The research was conducted between 2021 and 2023. During the COVID-19 pandemic (2021 and January–February 2022), research was conducted only when sanitary restrictions were not in place, i.e., when training and sports competitions were permitted. The criteria for inclusion in the study were being at least 18 years of age and practising judo or another sports discipline for at least 1 year. On the other hand, pregnancy or previous childbirth within the last year were criteria for exclusion from the study. No other health-related inclusion or exclusion criteria were applied because the study was conducted in sports clubs on the occasion of training or sports competitions and during national team training camps. According to Polish law, athletes in full health are allowed to participate in professional sport activities (training, sports competitions). Owing to a connection between the occurrence of urinary incontinence and frequent jumping shown in the studies, representatives of such sports as volleyball, basketball, the long jump, triple jump, high jump and gymnastics did not qualify for the control group [24, 25]. Similarly, because of the inconclusive findings concerning the impact of running on urinary incontinence, the studies did not include women who were professional runners, regardless of the distance usually covered [19, 25]. In total, the fully completed questionnaires of 88 women practising professional judo were collected. The control group was finalised after the completed questionnaires from 88 women were obtained. The characteristics of the studied groups and the homogeneity of the groups are presented in Table 1. In the control group, 48 women practised individual sports disciplines (canoeing, cycling, fencing, skating, swimming, table tennis, triathlon and weightlifting) and 40 practised team sports (football, handball and ice hockey). The number–percentage distribution of the studied women from the control group according to the practised sports discipline is presented in Supplementary Table 1.

**Table 1:** Demographic data of the participants (mean, SD, range)

Characteristics	Judo group (*n* = 88)	Control group (*n* = 88)	*p* value
Age (years)	21.63 (3.3) 18–35	22.44 (3.3) 18–33	0.107*
Weight (kg)	63.7 (9.5) 39–89	62.97 (8.1) 49–97	0.577*
Height (cm)	165.98 (6.6) 148–183	167.5 (6.1) 155–186	0.104*
BMI	23.03 (2.5) 16.98–29.06	22.44 (2.6) 17.36–32.41	0.126*
Childbirth (*n*)	1	3	0.341**
Sports career (years)	12.77 (3.6) 4–24	10.55 (3.7) 1–20	0.001*
Training frequency (number/week)	7.7 (2.8) 2–20	6.5 (3.4) 2–16	0.01*
Number of training hours (h/week)	14.0 (5.4) 4–30	12.6 (5.3) 3–30	0.075*

*t test

**Chi-squared test

The research was conducted using anonymous questionnaires. The questionnaires were distributed during training sessions, sports competitions and training camps. Printed, anonymous questionnaires in the form of a clipped set, consisting of a cover letter explaining the circumstances and purpose of the study, a questionnaire concerning socio-demographic data and several other questionnaires (Baecke, SF-36, FKB-20 and KCS), including a questionnaire assessing the occurrence of LUTS, were distributed in a white envelope with return address stamps or in an unstamped white envelope for questionnaires that could be returned to a collection container for completed questionnaires. The questionnaire assessing LUTS was consciously placed in the set of questionnaires and a cover letter indicated the self-assessment of the health-oriented quality of life of women athletes as the aim of the study, so as not to overly focus the attention of the surveyed women on lower urinary tract issues. For this reason, the questionnaire assessing LUTS was never placed first in the set of questionnaires and after every fifth set, its order was changed by one place in the set.

The study did not specify the time required to complete the questionnaires—they could be returned on the same day, on the next day, or else returned by post.

Every third person, after returning a set of questionnaires in a sealed envelope, was given a second set in a self-addressed envelope, asking them to complete them 4 weeks after the first set was returned. For subsequent statistical analyses, these sets were coded numerically, allowing for subsequent pairing of Core Lower Urinary Tract Symptom Score (CLSS) questionnaires. A total of 64 such sets were distributed, with 53 returned. This procedure allowed for an assessment of the reliability of responses to the Polish version of the CLSS questionnaire.

The CLSS questionnaire was used to assess the presence of LUTS [26]. The CLSS questionnaire is designed to be completed independently by the respondents and includes an assessment of the most important symptoms related to bladder and lower urinary tract functioning. The CLSS questionnaire took the form of a one-page printed table, which consisted of a total of 11 questions. The questionnaire included 10 questions about LUTS, i.e., increased frequency of urination during the day (question 1), nocturia—frequency of urination at night (question 2), sudden and strong urge to urinate (question 3), urine leakage because of the inability to hold back (question 4), urine leakage when coughing, sneezing or straining (question 5), urinating in a slow stream (question 6), urinating with effort (question 7), a feeling of incomplete bladder emptying after urination (question 8), bladder pain (question 9) and urethral pain (question 10). Answers to these questions were scored on a scale of 0 to 3 points. The final, 11th question concerned the respondent’s assessment of their satisfaction with the current condition of their urinary tract and was scored on a scale of 0 to 6 points [26].

The characteristics of the participants were described by the mean and standard deviation. Using Slovin’s formula, the minimum sample size was calculated, taking into consideration the original population size of women who practised competitive sports in Poland at the time of the study. Based on the total number of 53,503 women practising competitive sports in Poland, the minimum number of participants should be 156. The total of 176 competitive female athletes participated in the study and therefore met these minimum requirements. The interclass correlation coefficient (ICC) was used to calculate the reliability of Polish version of the CLSS questionnaire. The internal consistency of the questionnaires was assessed using Cronbach’s alpha coefficient. The occurrence of pelvic floor dysfunction symptoms was presented numerically and as a percentage within the groups. The occurrence of symptoms and their intensity were described by the mean, standard deviation and 95% CI. The differences in demographic parameters (age, height, weight, BMI, mean number of years of sports career, training frequency and number of hours of training) and scores of CLSS were analysed using a *t* test for independent samples or by the Mann–Whitney test since the data did not show a normal distribution. Differences in symptoms were analysed by the Chi-squared test. The level of significance was set at *p* < 0.05.

## Results

The CLSS questionnaires were initially administered to 192 competitively active women (95 judoists and 97 other sportswomen). Of the 95 competitive judoists, 2 returned blank questionnaires and 5 returned incomplete responses. Ultimately, 88 fully completed CLSS questionnaires were analysed. From the group of women practising other sports, 9 participants returned incomplete CLSS questionnaires, resulting in a formation of an equal-sized control group. A total of 91.67% of surveyed female athletes were included in the analyses. However, of the 53 returned questionnaires completed after 4 weeks, 3 had missing responses and ultimately, 50 paired sets of CLSS questionnaires were used to calculate the ICC coefficient.

The Cronbach’s alpha internal consistency coefficient for the overall CLSS questionnaire score was 0.796 (95% CI 0.733–0.848). The α coefficient for individual questions in the CLSS questionnaire ranged from 0.739 (question 8) to 0.812 (question 10). As the obtained α coefficient values were > 0.7, the internal consistency of the Polish version of the CLSS questionnaire should be considered acceptable. Analysis of the results of the ICC (ICC_1.1_) showed a high degree of reliability for the repeatability of the overall CLSS questionnaire score (0.92; 95% CI 0.87–0.95). However, for the level of acceptance of the condition of CLSS, the repeatability of the results ranged from moderate to high (ICC_1.1_ = 0.77; 95% CI 0.62–0.86). The results of this analysis indicate the usefulness of the Polish version of the CLSS questionnaire in population studies of women practising sports professionally in Poland.

**Fig. 1:**
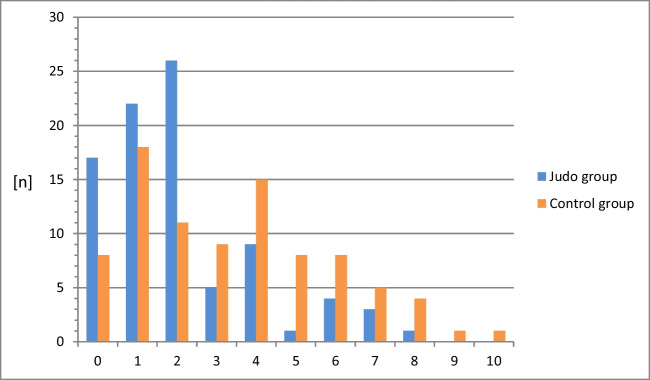
Number of lower urinary tract symptoms (LUTS) among female judo athletes and women from the control group

**Fig. 2:**
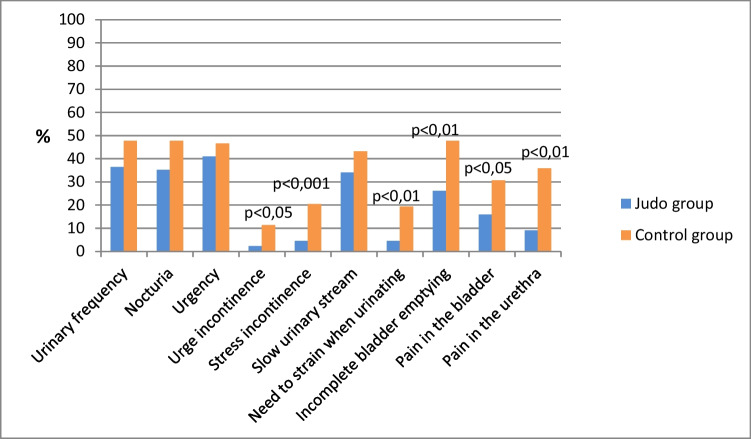
Occurrence of lower urinary tract symptoms (LUTS) according to the surveyed groups

Among 88 female judo athletes, 17 competitors (19.31%) did not indicate any presence of LUTS (Fig. 1). The majority of the remaining 65 female judo athletes (67.6%) experienced one (22 women) or two (26 women) symptoms. Only one female athlete reported eight symptoms. Among sportswomen from the control group 8 athletes had no symptoms of LUTS (9.09%) and from the remaining 80 women from this group 42 athletes (52.5%) indicated the presence of at least four symptoms. 1 sportswoman (1.13%) indicated the presence of the maximum number of symptoms (10 symptoms) included in the CLSS questionnaire. The average number of symptoms from LUTS in the judo and the control group women was statistically significantly higher in the control group (Table 2). Women practising judo most often indicated the occurrence of the urgency symptom (40.9%), an increased number of daily micturition (36.36%) incidents and night micturition (35.22%; Fig. 2). However, these symptoms were rare, as the maximal intensity of daytime micturition (15×) was only indicated by 2 female judo athletes and 1 female judo athlete indicated the maximal intensity of nocturnal micturition (4×). Among athletes from the control group the most frequent symptoms were the increased number of daily micturitions (47.72%), incomplete bladder emptying (47.72%), increased nocturnal micturitions (47.72%) and a slow urinary stream (43.18%). The control group was also characterised by a mild intensity of symptoms, with only 4 participants indicating the frequent occurrence of incomplete bladder emptying and 3 indicating the maximum frequency of daily micturition. The occurrence of incontinence symptoms was indicated by a total of 6 female judo athletes, 2 of whom suffered from urgency incontinence symptoms and 4 suffered from stress incontinence symptoms. These symptoms were rare and only 1 athlete indicated that she was sometimes experiencing the symptoms of stress incontinence. In the control group, 10 athletes (11.36%) indicated the occurrence of urgency incontinence symptoms and 18 (20.45%) experienced the occurrence of stress incontinence symptoms. Similar to the case of female judo athletes, these symptoms occurred sporadically, as only 1 woman indicated that the symptoms of urgency incontinence occurred frequently and 5 athletes indicated that the symptoms of stress incontinence occurred sometimes. The Chi-squared test showed that only in relation to the symptoms of frequency of daily and nocturnal micturition, the symptoms of a sudden, strong, difficult-to-stop urge to urinate and the symptoms of a slow urinary stream, no statistically significant differences were found between the groups. Among 10 analysed symptoms, the score of 3 of them (nocturia, incomplete bladder emptying after urination and urethral pain) were statistically significantly less frequent among women practising judo. The mean of the overall CLSS score was statistically significantly higher among sportswomen from the control group (Table 3).

**Table 2:** The number of symptoms and the level of acceptance of the condition of the lower urinary tract (mean, SD, 95% CI) in both groups of surveyed women

	Judo group	Control group	*p* value
Number of symptoms	2.07 (1.9) 1.67–2.48	3.42 (2.4) 2.9–3.94	0.001*
The level of acceptance of the condition of Core Lower Urinary Tract Symptom Score	0.93 (1.1) 0.67–1.18	1.52 (1.5) 1.19–1.84	0.01*

*Mann–Whitney *U* test

**Table 3:** Lower urinary tract symptoms intensity level and general Core Lower Urinary Tract Symptom Score (CLSS) score (mean; SD, 95% CI) in both groups of surveyed women

Symptom	Judo group	Control group	*p* value
Daytime frequency	0.46 (0.7) 0.31–0.61	0.75 (0.9) 0.55–0.94	0.067*
Nocturia	0.39 (0.5) 0.27–0.52	0.6 (0.7) 0.44–0.75	0.041**
Urgency	0.46 (0.6) 0.33–0.59	0.61 (0.7) 0.45–0.76	0.147**
Urge incontinence	0.02 (0.1) −0.009–0.05	0.13 (0.4) 0.04–0.22	0.296*
Stress incontinence	0.05 (0.2) −0.002–0.11	0.26 (0.5) 0.14–0.37	0.067*
Slow stream	0.43 (0.6) 0.29–0.57	0.56 (0.7) 0.41–0.72	0.192**
Straining	0.04 (0.2) 0.001–0.08	0.28 (0.6) 0.15–0.42	0.081*
Incomplete emptying	0.34 (0.6) 0.2–0.48	0.69 (0.8) 0.51–0.87	0.0078*
Bladder pain	0.2 (0.5) 0.09–0.31	0.45 (0.7) 0.29–0.61	0.073*
Urethral pain	0.1 (0.3) 0.03–0.17	0.43 (0.7) 0.26–0.59	0.028*
CLSS Score	2.53 (2.6) 1.96–3.1	4.79 (4.1) 3.9–5.68	0.000063*

*Mann–Whitney *U* test

**t test

To summarise the results of this study, 92.05% of all female judo athletes expressed a positive opinion (delighted, pleased, mostly satisfied) about the current condition of their lower urinary tract. In the control group, the percentage of respondents similarly assessing the condition of their urinary tract was 73.86%. Negative feelings related to the condition of their lower urinary tract were expressed by 4 women practising judo (4.54%) and 10 (11.36%) surveyed women from the control group. Mixed feelings were expressed by 3.41% of the female judo athletes and 14.77% of women from the control group. 95% CI of the level of acceptance of the condition of CLSS in the group of sportswomen practising judo was 0.67–1.18, in the control group it was 1.19–1.84, and the differences found were statistically significant (Table 2).

## Discussion

Studies concerning the prevalence of LUTS in women athletes are practically limited to assessing the presence of UI symptoms. In these analyses, only three papers included women who practised professional judo [1, 18, 27]. Parmigiano et al. [27] determined the frequency of UI in female judo athletes to be 33.3%, but their study focused on adolescent female athletes (mean age 15.4 years ±2 years). Rodríguez-López et al. [18] estimates the prevalence of UI among judo athletes to be at 16.3% and it is at a lower level than in representatives of other combat sports (boxing—31.3%, karate—45.5%) and slightly higher than in taekwondo athletes (15.4%). Unfortunately, these data concern 49 judo athletes, both male and female. In the study by Almeida et al. [1], the prevalence of UI among sportswomen practising judo was estimated to be quite high at 44.4%, the vast majority of whom suffered from SUI symptoms (75%). Unfortunately, this study is not very reliable because the analysis involved only 9 female athletes (4 with UI symptoms of unknown severity). The results of our study are convergent only with regard to the ratio of the occurrence of SUI symptoms to urge urinary incontinence (UUI) symptoms (75% vs 25%). In the studied population of 88 female judo athletes, only 2 athletes indicated the rare occurrence of UUI symptoms, whereas 4 athletes had SUI symptoms (3 rarely and 1 sometimes). Regardless of the large differences in the number of the surveyed female judo athletes, the approach to the analysed phenomenon is also radically different. In the study by Almeida et al. [1] cited above, classifications into respondents with or without UI symptoms were based on the question “Do you experience an involuntary loss of urine?” and a “positive” answer was followed by further questions about the frequency of urine loss, loss scenarios and strategies to control urinary loss. A significant percentage of adult healthy women can give an affirmative answer to the first question formulated in such a way. In our study a CLSS questionnaire was used, in which the first two questions had an emotionally “indifferent” character, as they concerned the frequency of daytime and night-time micturition. The occurrence of the next eight symptoms is summarised by one question, “How often do you have the following symptoms?”, and the questions regarding UUI and SUI (items 4 and 5) are “Leaking of urine because you cannot hold it” and “Leaking of urine, when you cough, sneeze, or strain” [26]. On the one hand, this does not result in a focus being placed on one problem alienated from the whole, and on the other hand, it defines more precisely the symptoms of UUI and SUI. An important difference was also related to the data collection method. In our study, the CLSS questionnaire was one of several questionnaires. The surveyed female judo athletes were informed that the purpose of the study was to assess their current health condition in the context of the sports discipline practised by them. In the set of questionnaires, the CLSS questionnaire never appeared first and its order in the set was changed after a few respondents. In this way, in our opinion, we minimised the excessive focus on one health problem. In our view, targeting respondents with questions that directly address a health problem, such as UI, may have negative cultural connotations and may result in the display of a dual approach to the problem, overemphasising one’s health problems based on a sense of injustice, or ignoring the problem in the form of a kind of self-deception and related denial of the existence of a specific health issue. A relatively long time ago this problem was noted by Sandvik et al. [28], when confronting the diagnosis made on the basis of interviews concerning urodynamic tests. The percentage of SUI increased from 51 to 77% and UUI decreased from 39 to 11%. In other urodynamic tests conducted on nulliparous physical education students, 6 of the 7 showed evidence of stress incontinence [29]. This fact suggests a cautious interpretation of the obtained data concerning the prevalence of UI. In our opinion, the problem of UI in women’s professional sport is somewhat exaggerated, especially when data concerning the severity of symptoms are taken into account. In many works, the problem of UI is presented on a “0—1” basis (“no—yes”), and in those results, where information concerning the UI severity is included, the majority of surveyed sportswomen choose terms such as “slight,” “rarely,” “few drops,” “few times,” and “less than twice/week” [16, 18, 19, 30]. With such a diverse methodology used to obtain results and with the tendency to pass clinical experience on to professional sports, there are notable tendencies to stigmatise certain sports such as volleyball.

To the best of our knowledge, this study is the first one to date that was used to analyse the occurrence of symptoms other than UI of the lower urinary tract in judo practitioners. We decided to compare female judo athletes with women professionally practising other sports disciplines. The control group consisted of women practising 12 different sports disciplines (40 women practising team sports and 48 women practising individual sports). The group included women practising both indoor and outdoor sports with different characteristics of physical effort during the sports competition, as well as a different specificity of the training. Against this background, the results produced by female judo athletes clearly differ from those of the control group. Out of the 10 symptoms analysed, no statistically significant differences were found only in the case of the occurrence of four symptoms. The remaining six analysed symptoms of LUTS were found statistically less frequently in professional judo athletes. The better condition of the lower urinary tract in women practising judo was confirmed by the statistically significantly lower number of symptoms, lower severity of 3 (out of 10) analysed symptoms and a lower CLSS score. The level of self-esteem regarding the condition of the lower urinary tract in sportswomen from the judo group was statistically significantly better. Other factors had the greatest impact on the latter assessment in judo athletes. The level of acceptance of the condition of the respondents’ lower urinary tract depended mainly on the severity of pain in the urethra and bladder and on the overall score of CLSS, whereas the need to strain when urinating, incomplete bladder emptying, slow urinary stream, urge incontinence, urgency and a CLSS score had the greatest influence on a similar assessment among women practising other sports disciplines.

The results of the assessment of the condition of the lower urinary tract in female judo athletes are puzzling. Judo is characterised by high-intensity physical strain, both during training and during competitions. Training sessions are most often performed in pairs and include a repetition of techniques without throwing (“uchi-komi”), a repetition of throwing to perfect the technique (“nage-komi”), and sparring bouts to enforce techniques under fight-practice conditions (“randomi”) [20]. The effort required during a sports fight in judo is considerable and involves the performance of a whole range of complex motor patterns in standing and groundwork positions [31]. The specificity of this discipline therefore favours such motor characteristics as strength, endurance, coordination, speed, agility and flexibility. The combination of strength and endurance on the one hand, with speed and agility on the other, requires the perfect control of one’s own body, and this is inextricably linked with the optimal control of the pelvis and trunk, thus allowing one to control one’s center of gravity even when losing balance (after all, the “adventure” in judo starts with learning how to fall). Perhaps the secret of the “more efficient” urinary tract in professional female judo athletes lies in the long-term shaping of such motor patterns that improve both the co-contraction between the so-called deep stabilisers of the trunk and the pelvic floor muscles, as well as paving the way for the preactivation of the latter. Perhaps this sports discipline favours female athletes who are characterised by a better core stability and this is the group that continue their sport career. In simple terms we may say that a judo athlete requires a stable torso and fast arms and legs. In order to confirm a qualitatively more efficient mechanism of motor control in the pelvis and trunk in female judo athletes, it is necessary to study the mutual feedback between the PFM functioning and the so-called local stabiliser of the lower part of the trunk.

The strength of our study is the number of analysed female judo athletes practising judo at a high level (the study also included national team athletes, including participants in the recent Tokyo Olympics). A positive aspect of this study is the evaluation of the occurrence of various LUTS rather than a focus on only one selectively chosen symptom. Another positive aspect of this study is the use of the CLSS questionnaire. It is a simple form, and the way in which the questions are formulated together with the fact that it is not time-consuming to complete, make this questionnaire very useful for screening studies of professional athletes. The limitation of our study is that it does not cover other aspects of pelvic floor dysfunction. The failure to include the occurrence of bowel, prolapse and sexual symptoms in our study does not allow us to clearly assess the long-term effect on the female pelvis when practising professional judo. Another limitation is the small number of women who gave birth. This limited the scope of this study, which prevented a more detailed analysis of the impact of childbirth on the occurrence of LUTS symptoms.

## Conclusions

The results of our study indicate that, in comparison with other sports disciplines, professional judo is not a risk factor in the occurrence of increased LUTS. The most frequent symptom among female judo athletes is urgency (40.09%), but in the vast majority of the cases it occurs quite rarely. The level of health satisfaction among women practising judo concerning the condition of the respondents’ lower urinary tract is high.

## Supplementary Information

Below is the link to the electronic supplementary material.

Supplementary file1 (DOCX 34 KB)

## Data Availability

Restrictions apply to the datasets. The datasets presented in this article are not readily available because the data are part of an ongoing study. The data that support the findings of this study may be available from the corresponding author upon reasonable request.
