# Supplementary material for: Assessing Lower Urinary Tract Symptoms in Women Practising Competitive Judo: Findings from a Cross-sectional Study
Source: Int Urogynecol J. 2026 Feb 27;37(6):1699–707. doi: 10.1007/s00192-026-06523-9 (PMC13309410; doi:10.1007/s00192-026-06523-9)
Supplement: Supplementary file 1 — Supplementary file1 (DOCX 34 KB) [file 192_2026_6523_MOESM1_ESM.docx]

**Assessing Lower Urinary Tract Symptoms in Women Practising Competitive Judo: Findings from a Cross-sectional Study**

Supplementary Table 1. Type of sports discipline practised in the control group.

| Sports type | No. of subjects (%) |
| --- | --- |
| Football | 16 (18.18) |
| Handball | 13 (14.77) |
| Ice hockey | 11 (12.5) |
| Swimming | 11 (12.5) |
| Cross-country cycling | 8 (9.09) |
| Road cycling | 7 (7.95) |
| Weightlifting | 7 (7.95) |
| Canoeing | 6 (6.81) |
| Fencing | 4 (4.54) |
| Table tennis | 3 (3.4) |
| Skating | 1 (1.13) |
| Triathlon | 1 (1.13) |

Supplementary Fig. 1. Feelings of the respondents as they related to the assessment of the current condition of the lower urinary tract.
